# Investigating the Feasibility and Performance of Hybrid Overmolded UHMWPE 3D-Printed PEEK Structural Composites for Orthopedic Implant Applications: A Pilot Study

**DOI:** 10.3390/bioengineering11060616

**Published:** 2024-06-17

**Authors:** James A. Smith, Cemile Basgul, Bita Soltan Mohammadlou, Mark Allen, Steven M. Kurtz

**Affiliations:** 1 Implant Research Core, School of Biomedical Engineering, Science and Health Systems, Drexel University, Philadelphia, PA 19104, USA; cb997@drexel.edu (C.B.); smk38@drexel.edu (S.M.K.); 2 Department of Mechanical Engineering, Drexel University, Philadelphia, PA 19104, USA; bs3298@drexel.edu; 3 Orthoplastics, Bacup OL13 9EF, UK; mallen@orthoplastics.com

**Keywords:** polyetheretherketone, ultra-high molecular weight polyethylene, porosity, material extrusion, hybrid manufacturing, 3D printing, additive manufacturing

## Abstract

Ultra-high-molecular-weight polyethylene (UHMWPE) components for orthopedic implants have historically been integrated into metal backings by direct-compression molding (DCM). However, metal backings are costly, stiffer than cortical bone, and may be associated with medical imaging distortion and metal release. Hybrid-manufactured DCM UHMWPE overmolded additively manufactured polyetheretherketone (PEEK) structural components could offer an alternative solution, but are yet to be explored. In this study, five different porous topologies (grid, triangular, honeycomb, octahedral, and gyroid) and three surface feature sizes (low, medium, and high) were implemented into the top surface of digital cylindrical specimens prior to being 3D printed in PEEK and then overmolded with UHMWPE. Separation forces were recorded as 1.97–3.86 kN, therefore matching and bettering the historical industry values (2–3 kN) recorded for DCM UHMWPE metal components. Infill topology affected failure mechanism (Type 1 or 2) and obtained separation forces, with shapes having greater sidewall numbers (honeycomb-60%) and interconnectivity (gyroid-30%) through their builds, tolerating higher transmitted forces. Surface feature size also had an impact on applied load, whereby those with low infill-%s generally recorded lower levels of performance vs. medium and high infill strategies. These preliminary findings suggest that hybrid-manufactured structural composites could replace metal backings and produce orthopedic implants with high-performing polymer–polymer interfaces.

## 1. Introduction

Insert overmolding is an advanced manufacturing technique that enables the bonding of two chemically contrasting materials by injecting one material into a pre-made insert of another at optimized temperature, pressure, and time conditions [1,2]. Post-production, such multi-material constructs are then further purposed to produce “gold-standard” medical devices such as those found in patella implants. Traditionally, these are in the form of metal–polymer interfaces such as titanium/cobalt chrome alloy-ultra-high-molecular-weight polyethylene (UHMWPE). However, metallic components are expensive and have a higher elastic modulus than cortical bone, which causes stress shielding and has raised concerns about metal release into surrounding tissues [3,4,5,6]. Polymer–polymer interfacial constructs offer an alternative whereby they are less expensive to manufacture, have a closer modulus to bone, and are chemically inert [7,8]. Despite overmolding’s prowess, stress concentrations typically arise at the adjoined material interface, which, over time, may lead to device failure and revision surgery. Efforts to enhance interfacial connectivity have been explored; however, there is still room for improvement [9,10,11,12,13,14,15,16,17,18,19]. Additive Manufacturing (AM) is a maturing fabrication technology that can produce complex geometries with unmatched levels of biomimicry, topology, and strength-to-weight ratios such as Lattices and Triply Periodic Minimal Surfaces (TPMS) [20,21,22]. The topological features can be readily altered to optimize biological, mechanical, and physical behavior for end application [23,24,25]. Hence, studies investigating the overmolding of AM lattices to improve mechanical interlocking are beginning to be explored [24,25,26,27,28,29,30]. For example, Verma et al. [24] investigated the effect of strut size (low and high) and lattice body-centered cubic with z-strut (BCCZ), face-centered cubic (CFCC), and truncated octahedron (Kelvin) types on the adhesion performance of selective laser-sintered 316 L stainless steel bonded to a polymer resin. Ding et al. [29] assessed the effect of strut angle (30°, 45°, and 60°) in a truss lattice design, as well as the effects of TPMS lattice (diamond, gyroid, and neovirus) design and porosity (60%, 76%, and 84%) on the interlocking strength between material-jetted UV-curable acrylate and polydimethylsiloxane (PDMS). Zou et al. [30] explored the lap shear strength of overmolded UHMWPE titanium (Ti6Al4V) (produced by electron beam-melted (EBM)) specimens with different porous interfaces (non-structured (dense), diamond, and honeycomb) and Alhmoudi et al. [31] evaluated the interfacial adhesion between acrylonitrile butadiene styrene (ABS) directly extruded onto and an aluminum alloy. Despite the aforementioned studies, there is a lack of literature detailing the ideal porous topologies and surface features to maximize bonding strength between contrasting medical-grade polymer–polymer structures.

Polyetheretherketone (PEEK) is a clinically favored polymer used in a variety of “gold-standard” medical devices and implants [32,33,34,35,36]. Consequently, efforts to extrude the polymer have gained traction due to the availability of certified printers and filaments. However, PEEK’s intrinsic properties (melting temperature, viscosity, and semi-crystallinity) make it a challenge to print for optimal mechanical, geometrical, and aesthetic tolerances, requiring print parameters to be constantly monitored between builds [32,37,38,39,40,41,42,43,44,45]. Regardless, PEEK’s clinical familiarity and performance make it a promising candidate to be overmolded with another biomedically relevant polymer, UHMWPE [46]. 

In this study, we ask the following research questions: (1) Can hybrid structural UHMWPE–PEEK composites be successfully fabricated by industry overmolding standards and additive manufacturing? (2) Can hybrid structural composites match the requirements of metal metal-backed UHMWPE components? (3) How do hybrid UHMWPE–PEEK specimens fail under tensile loading? (4) Are there trends associated with different topologies (shape) and feature sizes (infill-%) vs. separation forces in UHMWPE–PEEK constructs?

## 2. Materials and Methods

### 2.1. Designing and Printing of Porous Test Specimens

**Figure 1:**
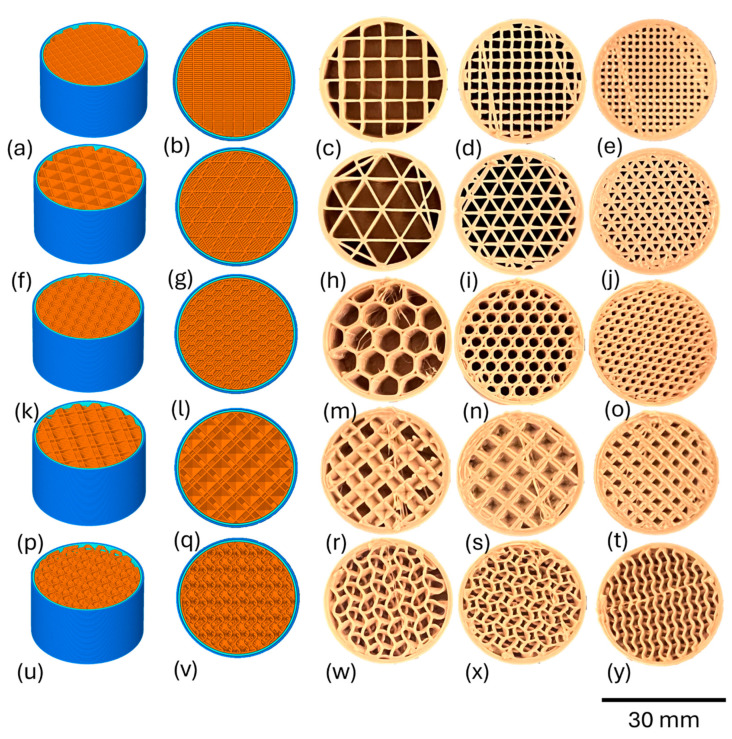
Grid topside (**a**) and top-down (**b**) digital profiles. 20 (**c**), 40 (**d**), and 60 (**e**) infill-% printed porous PEEK. Triangular topside (**f**) and top-down (**g**) digital profiles. 20 (**h**), 40 (**i**), and 60 (**j**) infill-% printed PEEK. Honeycomb topside (**k**) and top-down (**l**) digital profiles. 20 (**m**), 40 (**n**), and 60 (**o**) infill-% printed PEEK. Octahedral topside (**p**) and top-down (**q**) digital profiles. 30 (**r**), 40 (**s**), and 60 (**t**) infill-% printed porous PEEK. Gyroid topside (**u**), and top-down (**v**) digital profiles. 20 (**w**), 25 (**x**) and 30 (**y**) infill-% printed porous PEEK.

Cylindrical specimens (height = 18.5 mm and Ø = 30 mm) were designed in CAD software (Fusion 360, V 2.0.9719, Autodesk, San Rafael, CA, USA) and sliced via Simplify 3D software (Version V4/V5, USA). Grid, triangular, or honeycomb porous infill shapes with varying infill densities (20%, 40%, and 60%) were implemented into the top third of the cylinder, which were 6 mm in depth, while the rest remained dense (rectilinear-100% infill) (Figure 1).

Two additional infill strategies, octahedral and gyroid, were explored with 30%, 40%, and 60% and 20%, 25%, and 30% infills, respectively (Figure 1). The infill ratios per design were determined by printability considerations, with a focus on maintaining uniformity in appearance. The specimens were printed under identical print parameters (Table 1).

**Figure 2:**
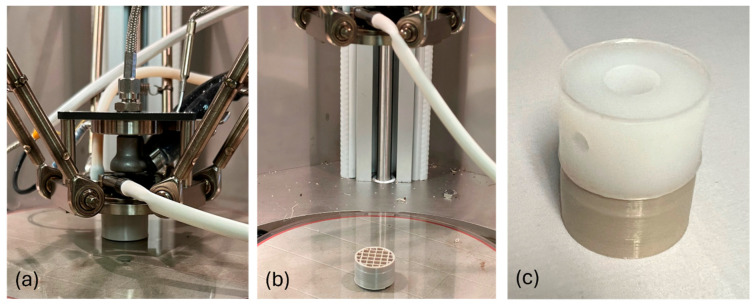
Grid-20% porous PEEK specimen during (**a**) and post-printing (**b**) and post-UHMWPE overmolding and drilling (**c**).

All specimens (n = 5) were printed in the center of the build platform on a medical material-extrusion printer (EXT 220 MED, 3D systems, Munich, Germany) with a PEEK filament (Ø = 1.75 mm, M40, Evonik, Essen, Germany) (Figure 2a,b).

### 2.2. Overmolding and Mechanical Testing

The AM PEEK specimens were overmolded with GUR 1020 UHMWPE powder at industrially relevant DCM conditions (Figure 2c). The bottom (PEEK), sides, and top (UHMWPE) of the specimens were drilled to create necessary fixation points for mechanical testing. The overmolded specimens (n = 3) were loaded onto a uniaxial testing machine (H10KS, Tinius Olsen, Surrey, UK). The PEEK region was fixated into a specimen holder using an M6 screw, while the UHMWPE portion was attached via a 4 mm Ø cross pin in a 10 mm dowel. The setup was designed to determine the separation forces required to detach the two contrasting polymers at their interfaces. Specimens were then loaded at 1 mm/min with a load cell of 10 kN in the positive direction, i.e., tension.

### 2.3. Cross-Sectional Analysis

A non-mechanically tested, intact, overmolded UHMWPE–PEEK specimen (n = 1) from each topology group and infill-% was cross-sectioned for investigation using an optical microscope (Vision Lynx, Woking, UK) at 6× magnification.

### 2.4. Micro-CT Analysis

Complete honeycomb and gyroid overmolded specimens (n = 1 each) were examined for internal structures by micro-CT (Zeiss Xradia 620 Versa system, Carl Zeiss, Dublin, CA, USA). The system was set to operate at a voltage of 70 kV and a power of 8 W, achieving a spatial resolution of 12.93 µm using a 0.4× detector. Throughout the scanning process, 1602 projection images were collected, and LE1 and LE2 filters were used to reduce noise and enhance image clarity. Following data acquisition, the raw projection data were processed using Zeiss XRM reconstructor software (V. 2022.1, Dublin, CA, USA), which applied standard corrections for center shift and beam-hardening to ensure precise imaging. Image refinement was further enhanced by Gaussian filtration, which enhanced the quality of segmentation. The segmentation process utilized ORS Dragonfly Pro software (V. 2022.1, Dublin, CA, USA), which employed a thresholding technique with manual adjustments to increase accuracy. Finally, a quantitative analysis was conducted to evaluate the infill-shape surface area (S/A) (%), strut thickness, voids, and distribution.

## 3. Results

### 3.1. Mechanical Testing

The mechanical performance of the UHMWPE–PEEK specimens is presented in Table 2. Separation forces were recorded in the range of 1.97–3.86 kN. Specimens with the lowest infill-% generally displayed the lowest levels of mechanical performance (e.g., Grid-40% and Grid-60% recorded +69.5% and +64.1% increases in pull-out force vs. Grid-20%) and the greatest rises in performance were witnessed between low and medium infill-% i.e., grid = +69.5%, triangular = +81.7% octahedral = +15.3% and gyroid = +11.6%. Octahedral was the exception, demonstrating an inverse relationship (e.g., Octahedral-30% recorded +17% and +25.1% increases in pull-out force vs. Octahedral-40% and Octahedral-60%). Grid and triangular specimens recorded the largest separation forces at medium infill-%, as opposed to honeycomb and gyroid, which benefited from the highest infill-% strategy. Rises in performance between medium and high infills-%s (for grid, triangular, octahedral, and gyroid) were recorded as +0.09%, substantially lower than those witnessed between low and medium (+44.5%) and low and high (+43.5%) infills. Infill shape appeared to have a direct effect on separation force, except between grid and triangular specimens.

**Figure 3:**
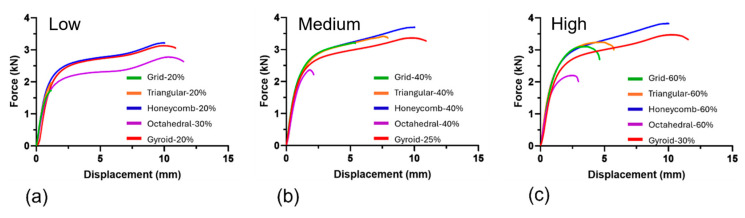
Separation forces of UHMWPE-porous PEEK specimens at low (**a**), medium (**b**), and high (**c**) infill-%.

Force vs. displacement graphs for the UHMWPE–PEEK are plotted in Figure 3. Grid-20% and Triangular-20% specimens displayed brittle-like behavior prior to failure, while Honeycomb-20%, Octahedral-30%, and Gyroid-20% saw similar-to-higher levels of elasticity, followed by high ductility until failure. At medium infill-%s, grid and triangular shapes exhibited a brittle–ductile profile far closer in behavior to equivalent honeycomb and gyroid structures. Octahedral-40% reverted to a brittle-like profile within rising infill-%. Grid-60% and Triangular-60% specimens displayed higher elastic behavior than at their lower and medium infill-%s; however, levels of ductility decreased vs. their respective 40% counterparts. Honeycomb-60% and Gyroid-30% displayed similar profiles to those seen at low and medium infill-%, while Octahedral-60% displayed lower levels of elasticity and minor increases in ductility prior to failure vs. Octahedral-40%. * = minimum separation force that the specimen can withstand due to Type 2 failure (see below for full explanation).

**Figure 4:**
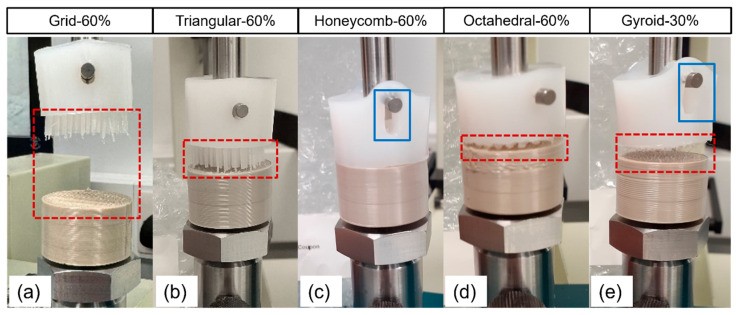
Overmolded UHMWPE-porous PEEK specimen during interfacial separation testing; Grid-60% (**a**), Triangular-60% (**b**), Honeycomb-60% (**c**), Octahedral-60% (**d**) and gyroid-30% (**e**). Red dashed boxes = Type 1 failure (separation at the polymer–polymer interfaces), and blue solid box = Type 2 failure (the fixation pin of the axial testing rig ripping through the UHMWPE).

Two general failure mechanisms were observed when assessing the overmolded samples: Type 1—separation at the polymer–polymer interface or Type 2—the fixation pin of the axial testing rig ripping through the UHMWPE block, in turn providing a minimum applied force that the polymer–polymer interface can tolerate, as opposed to an exact separation force (as obtained with polymer–polymer interfacial failure) (Figure 4). In some cases, a combination of both Type 1 and 2 failure mechanisms was seen during testing; however, the first failure always occurred in the UHMWPE and is therefore also classed as Type 2. All grid specimens failed by the separation of their interfaces (Type 1) irrespective of their infill percentages. The triangular specimens also displayed this trend; however, one specimen (Triangular-40%) witnessed failure within the UHMWPE portion. All Honeycomb-20% and 40% specimens failed in the UHMWPE section of the specimen (Type 2), while the majority (2/3) of the 60% specimens delaminated at the adjoining polymer interfaces. The majority of Octahedral-30% specimens experienced Type 1 failure. This trend continued for all Octahedral-40% and 60% specimens, respectively. All gyroid specimens (20%, 25%, and 30%) demonstrated Type 2 failure. There was no distinct relationship between force vs. displacement profiles and the recorded failure type.

### 3.2. Cross-Sectional Analysis

**Figure 5:**
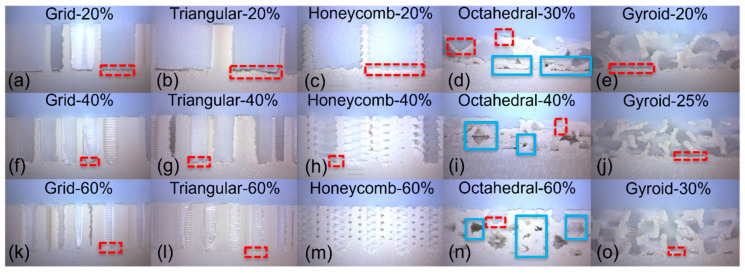
Cross-sections of UHMWPE-porous PEEK specimens. Grid-20% (**a**), Triangular-20% (**b**), Honeycomb-20% (**c**), Octahedral-30% (**d**), Gyroid-20% (**e**), Grid-40% (**f**), Triangular-40% (**g**), Honeycomb-40% (**h**), Octahedral-40% (**i**), Gyroid-25% (**j**), Grid-60% (**k**), Triangular-60% (**l**), Honeycomb-60% (**m**), Octahedral-60% (**n**), Gyroid-30% (**o**). Red dashed boxes = UHMWPE shrinkage, and blue solid boxes = pores in structure at 6× magnification.

Cross-sectional analysis revealed successful UHMWPE infiltration, irrespective of shape or infill-% (Figure 5). In grid, triangular, and honeycomb specimens, this was in the form of blocks of UHMWPE adhering to the base and sidewalls of the porous PEEK constructs. UHMWPE infiltration was far more limited in the octahedral specimens, especially with rising infill-%, and displayed greater numbers of unfilled cavities within porous regions (Figure 5d–n). Furthermore, these unfilled cavities suffered from structural collapse as a consequence of the overmolding process. Structural collapse was slightly observed for gyroid specimens; however, UHMWPE infiltration was complete throughout, irrespective of infill-%. Polymer shrinkage (an air gap between the polymer interfaces) was witnessed in all specimens, irrespective of infill shape or infill-%. Such phenomena were observed most in a low infill-% grid and triangular specimens and least in gyroid and honeycomb specimens.

### 3.3. Micro-CT Interfacial Analysis

**Figure 6:**
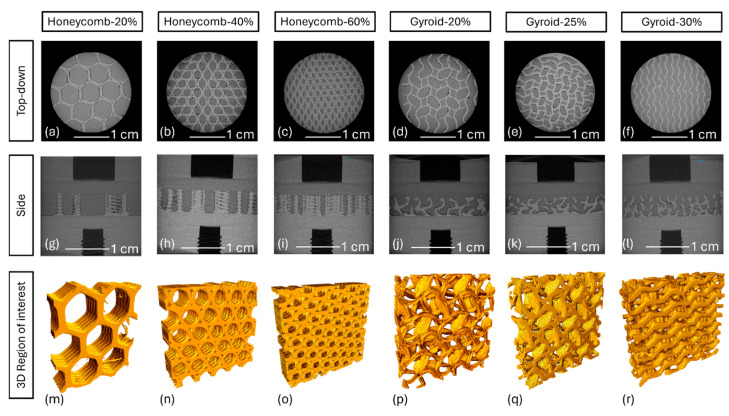
Micro-CT scans of UHMWPE-porous PEEK specimens. Top-down view of Honeycomb-20% (**a**), 40% (**b**), 60% (**c**) and Gyroid-20% (**d**), 40% (**e**) and 60% (**f**). Side-profile of Honeycomb-20% (**g**), 40% (**h**), 60% (**i**) and Gyroid-20% (**j**), 40% (**k**) and 60% (**l**). Three-dimensional region of interest of Honeycomb-20% (**m**), 40% (**n**), 60% (**o**), and Gyroid-20% (**p**), 40% (**q**), and 60% (**r**).

Micro-CT analysis confirmed that the printed honeycomb specimens had representative infill-%s compared to those selected in the slicing software (Figure 6 and Table 3), i.e., Honeycomb-60% obtained ~59% infill volume. Correspondingly, values for the overmolded UHMWPE were also considered representative. Greater deviations in PEEK and UHMWPE volumes were observed in the gyroid specimens, but the former and latter increased and descended in infill-% values as expected. Voids (areas of non-PEEK or UHMWPE) were observed up to 3%; however, this number was the smallest (0.6%) in the Honeycomb-60% specimens.

## 4. Discussion

Here, we present a hybrid manufacturing method comprised of overmolding UHMWPE onto a series of AM PEEK inserts with several different topologies and surface feature sizes to form biomedically relevant polymer–polymer interfaces for orthopedic applications. These composite structures were then tested to determine the separation forces required to cause failure. Separation forces were recorded as 1.97–3.86 kN, therefore matching and bettering the historical values (2–3 kN) recorded for DCM UHMWPE metal components (M. Allen, Orthoplastics “personal communication”, 12 April 2024).

Topology significantly influenced the separation forces observed in the UHMWPE–PEEK specimens, which was credited to S/A availability within the porous zones for UHMWPE to adhere, as well as the formation of interconnected networks between the porous infill shapes (i.e., gyroid). Higher levels of performance were observed with an increase in the number of internal side walls available for adherence, with honeycomb structures at all infill-%s exhibiting greater interlocking strength compared to triangular, grid, octahedral, and gyroid structures. Furthermore, the S/A of the internal walls increased further due to the formation of grooves between adjacent print layers, promoting additional UHMWPE friction and adhesion between the two polymers. S/A availability appeared to have a direct effect on the separation force and failure mechanism of the specimens, whereby those with fewer sidewalls (SW) to which to adhere recorded the lowest separation forces and generally displayed Type 1 failure across all infill levels e.g., triangular (3 SW) = 100%, octahedral (4 SW) = 89%, grid (4 SW) = 89%, honeycomb (6 SW) = 22% and gyroid (continuous SW + porous network) = 0% Type 1 failure. Similarly, Englert et al. [47] showed that higher S/A in their interlocking specimens aided polylactic acid (PLA) ingress and attachment to aluminum specimens, in turn reducing pull-out style failure in tension. 

In this study, octahedral specimens had tapered shallow pores vs. the other topologies. Hence, it is suspected that stress concentrations built up in the narrowest regions that the UHMWPE adhered to, i.e., the area of minimal contact area between UHMWPE and PEEK. Chueh et al. [48] also reported a negative correlation between strength and narrowing tapered angles/convex curvature, whereby such angles hindered polymer penetration in hybrid stainless steel and polyethylene terephthalate (PET) constructs. Furthermore, we observed greater numbers of unfilled pores/cavities for octahedral specimens under the microscope, which also contributed to poor separation forces, as there was less structural material to dissipate applied loads.

Gyroid shapes benefited from high contact areas for the UHMWPE to adhere to, as well as connected porosity in the x–y directions, enabling a complete polymer ingress network to be formed. As a result, none of the specimens failed at the interface, i.e., Type 2 failure, and recorded minimum separation forces. Exploration of the microstructure by microscope and micro-CT suggests that the generation of a porous network, along with increased channel depth, enhances the separation force of such components at all tested infill-%’s. This is credited to the network’s enhanced ability to distribute and resist the forces applied to their builds. This behavior is highly desirable and suggests that true separation forces for gyroid specimens should be higher than those initially reported, supporting even greater potential for the infill design strategy. In addition, the gyroid infill observed minor levels of the UHMWPE shrinkage from PEEK, which may contribute to enhanced levels of mechanical performance. Ding et al. [49] also observed that strength increased when PDMS was overmolded into acrylate 3D TMPS lattices (diamond, gyroid, and neovirus) vs. 3D truss lattices and 2D tooth pattern structures. This behavior was credited to enhanced levels of interlocking (due to the existence of porous networks), reducing stress concentration at the interface [49].

Specimens (grid, triangular, honeycomb, and gyroid) printed at medium and high infill-%s outperformed the structures printed with low infill percentages. This could be due to the bulk volume of the overmolded UHMWPE (not directly in contact with the PEEK walls) reducing with increasing infill-% and the distance between adjacent pores (in the z direction) decreasing. As a consequence, a greater number of individual porous regions exists within the interfacial region of the PEEK, enabling the load to be distributed more isotropically across the build vs. low infill-%s (as seen in microscopy and micro-CT images). Another contributing factor could be the forces from the overmolding process having a far greater effect on the adherence of the UHMWPE onto the PEEK, as more load is being placed on less surface area per pore. Additionally, this may also reduce the effects of UHMWPE shrinkage at the interface. Verma et al. [24] also reported higher separation strengths with increasing strut diameter in three different selective laser-sintered stainless steel lattices (body-centered cubic with z-strut (BCCZ), face-centered cubic (CFCC), and truncated octahedron (Kelvin)) interlocked with polymer resin. Combined average values for high and low strut density were reported as 65.3 and 56.2 MPa, respectively [24]. Octahedral specimens witnessed the opposite trend, whereby lower infill-%s outperformed both medium and higher levels. The rationale for this is the loss of advantageous surface features (such as deep pores and a semi-connected network along the x–y direction) with rising infill-%. Resultingly, UHMWPE could not ingress as deep or as interconnected through the porous PEEK, leading to poorer adhesion and performance. Ding et al. [29] confirmed the opposite effect, where the highest lap shear strength was achieved with high porosity (86%) vs. (low 60%) and medium (76%) in diamond TMPS lattices.

No difference was recorded between medium and low infill-%s for grid, triangular, honeycomb, and gyroid specimens. This may be due to all PEEK infill-%s offering similar levels of surface adhesion to the UHMWPE and the transmittance of overmolding forces minimizing interfacial shrinkage comparatively. Unlike the other test specimens, Honeycomb-60% specimens recorded 4 times fewer air voids than their low and medium counterparts, which may also contribute to the highest separation forces recorded. The formation of these air voids was credited to the overmolding process, whereby the UHMWPE was unable to fully ingress or shrank upon cooling [30,47]. Zou et al. [30] noted a similar phenomenon in overmolded UHMWPE AM titanium specimens, with honeycomb shapes recording lower levels of porosity vs. dense and diamond profiles, the latter of which suffered from air entrapment as a result of closed pore formations during printing [30]. Despite air voids existing in the UHMWPE–PEEK parts, separation forces still exceeded benchmark values without failure at the adjoining material interfaces and were comparable to levels (1.1–5.1%) witnessed in PLA-metal (AlSi10Mg) specimens [47]. Efforts should be made to optimize the overmolding process for AM inserts to minimize such air gaps in the future. For the most part, no significant differences in failure mechanism were seen with increasing infill-% in the specimens. Finally, none of the PEEK specimens experienced any surface feature or z-strength failure upon mechanical testing. Such a phenomenon is a typical weakness of material-extrusion-based prints due to the way the AM process builds components [50].

Lastly, we would like to note the study limitations. Five different topologies and three infill-% levels were explored; hence, our results are only applicable within these conditions. Three overmolded specimens were mechanically tested per print strategy, and hence, they may not be a true representation of a larger population. Larger sample sizes should be used to better represent the population and determine statistical significance between topologies and surface features in future studies. This also applies to both cross-sectional and micro-CT samples, which were limited to one per design. These preliminary designs were intended to explore infill strategies for 6-mm porous regions, which are typically deeper than those found in orthopedic devices. Efforts to reduce this depth should be considered in follow-up studies. Furthermore, alterations to the testing rig should also be investigated, e.g., the pin should have a larger diameter to prevent Type 2 failure and obtain exact separation forces for the specimens. Only a single set of build parameters was explored to print PEEK, and hence, optimized experimental designs may be useful in future studies to enhance mechanical performance, geometrical accuracy, and manufacturing output. The PEEK used in the study was short-contact grade (up to 30 days), although it is confirmed to be aligned with medical-grade standards. It is unclear whether specimens built on the EXT MED 220 are comparable to identical designs built on other printers. Washing and sterilization practices were not performed on the specimens. Hence, further studies are necessary to explore this, as washing and sterilization are prerequisites for implants. The current structures were tested in tension. Therefore, additional compressive, shear, compressive shear, and cyclic mechanical studies should also be performed as they represent real-life loading conditions of orthopedic implant components. Despite being professionally overmolded, specimens still presented regions of incomplete ingress (air voids) within the structure. Additional research to maximize the polymer ingress of AM PEEK inserts should be explored as greater separation forces could be achieved and then channeled into other medical applications. PEEK backings may be more susceptible to micro-movements than the titanium that they aim to replace. Hence, follow-up studies should be explored to assess pull-out strength post-osteointegration. Finite-element analysis (FEA) should also be integrated into future studies, helping to identify topologies and surface features that could promote better mechanical performance between the two polymers and further validate the obtained experimental findings.

## 5. Conclusions

Polymer–polymer interfacial components can overcome the drawbacks (cost, metal release, stress shielding, and medical imaging distortion) of pre-existing metal–polymer interfaces that are used in orthopedic devices. Furthermore, when coupled with additive-manufacturing technologies, such components further benefit from the geometrical freedom the technology allows. In this study, hybrid manufacturing methods were used to fabricate overmolded UHMWPE–PEEK specimens, which were mechanically tested to determine the effect of porous infill shape and infill-% on separation force values. Five topologies (grid, triangular, honeycomb, octahedral, and gyroid) and three levels of infill (low, medium, and high) were explored. Results suggest that shape, infill-%, and shape and infill-% impact the separation forces required to separate the two polymers. Honeycomb-60% recorded the highest levels of performance (3.86 ± 0.16 kN), beating pre-existing historical benchmarks (~2–3 kN) for metal–polymer DCM interface components. Performance was credited to high wall numbers within the porous zone to adhere to (increase in available surface area) and fewer air voids vs. the other test specimens. Furthermore, it is theorized that as infill-% rose, the forces applied during the overmolding process encouraged greater interfacial adhesion. Despite this, most specimens within this build are separated at the polymer–polymer interface. Gyroid-30% also bettered pre-existing metrics; however, the structure benefited from the formation of an interconnected porous structure in the x–y direction of the build. In turn, higher levels of UHMWPE ingress occurred throughout the build, forming a robust interface. Gyroid specimens all failed in the bulk UHMWPE, whereby the forces at the interface exceeded the capacity of the test fixture. Hence, the results obtained provide the minimum separation forces that such interfaces could tolerate, with true values expected to be higher. Efforts to reduce air voids within the structures should also aid in improving interfacial bonding and should be further investigated. This pilot study demonstrates the feasibility of hybrid-manufactured UHMWPE–PEEK structural composites, which could be translated into next-generation orthopedic implant components. 

## Figures and Tables

**Table 1:** Print parameters of porous PEEK specimens.

Printing Parameter	Porous PEEK Insert
Nozzle temperature (°C)	430
Printing speed (mm/s)	25
Printing speed of the first layer (% of the printing speed of subsequent layers)	100
Nozzle material	Brass
Nozzle diameter (mm)	0.4
Build platform material	Glass
Build platform temperature (°C)	250
Build chamber temperature (°C)	200
Layer thickness (mm)	0.3
First layer thickness (mm)	0.3
Build orientation (°)	90
Number of concentric perimeters	2

**Table 2:** Separation forces, strength, and failure mechanisms of overmolded UHMWPE-porous PEEK specimens.

Sample	Separation Force (kN)	Separation Strength (MPa)	Failure Mechanism
Grid-20%	1.97 ± 0.02	2.78 ± 0.02	Type 1
Grid-40%	3.33 ± 0.17	4.72 ± 0.24	Type 1
Grid-60%	3.23 ± 0.03	4.56 ± 0.05	Type 1
Triangular-20%	1.78 ± 0.05	2.52 ± 0.07	Type 1
Triangular-40%	3.24 ± 0.13 *	4.58 ± 0.18 *	Type 1 and 2
Triangular-60%	3.11 ± 0.03	4.4 ± 0.04	Type 1
Honeycomb-20%	3.23 ± 0.04 *	4.57 ± 0.05 *	Type 2
Honeycomb-40%	3.72 ± 0.1 *	5.27 ± 0.14 *	Type 2
Honeycomb-60%	3.86 ± 0.16 *	5.47 ± 0.23 *	Type 1 and 2
Octahedral-30%	2.22 ± 0.04 *	3.92 ± 0.06 *	Type 1 and 2
Octahedral-40%	2.37 ± 0.13	3.35 ± 0.18	Type 1
Octahedral-60%	2.22 ± 0.06	3.14 ± 0.08	Type 1
Gyroid-20%	2.99 ± 0.25 *	4.23 ± 0.36 *	Type 2
Gyroid-25%	3.34 ± 0.05 *	4.73 ± 0.07 *	Type 2
Gyroid-30%	3.46 ± 0.03 *	4.9 ± 0.04 *	Type 2

* = minimum separation force that the specimen can withstand due to Type 2 failure (see below for full explanation).

**Table 3:** Micro-CT data of overmolded UHMWPE-porous PEEK specimens.

Sample	PEEK Volume (%)	UHMWPE Volume (%)	Air Void Volume (%)
Honeycomb-20%	23.1	72	2.4
Honeycomb-40%	40.9	56.8	2.2
Honeycomb-60%	59	39.8	0.6
Gyroid-20%	24.6	71.5	3.2
Gyroid-25%	32.4	63.9	2.9
Gyroid-30%	33.3	63.4	3

## Data Availability

The raw data was provided with the upload of this manuscript.
